# Methods for acquiring MRI data in children with autism spectrum disorder and intellectual impairment without the use of sedation

**DOI:** 10.1186/s11689-016-9154-9

**Published:** 2016-05-05

**Authors:** Christine Wu Nordahl, Melissa Mello, Audrey M. Shen, Mark D. Shen, Laurie A. Vismara, Deana Li, Kayla Harrington, Costin Tanase, Beth Goodlin-Jones, Sally Rogers, Leonard Abbeduto, David G. Amaral

**Affiliations:** MIND Institute, UC Davis School of Medicine, 2805 50th Street, Sacramento, CA 95817 USA; Department of Psychiatry and Behavioral Sciences, UC Davis School of Medicine, Sacramento, CA USA; Imaging Research Center, UC Davis School of Medicine, Sacramento, CA USA

**Keywords:** Brain, Neurodevelopment, MRI, Compliance, Intellectual disability, Low-functioning autism, Applied behavior analysis

## Abstract

**Background:**

Magnetic resonance imaging (MRI) has been widely used in studies evaluating the neuropathology of autism spectrum disorder (ASD). Studies are often limited, however, to higher functioning individuals with ASD. MRI studies of individuals with ASD and comorbid intellectual disability (ID) are lacking, due in part to the challenges of acquiring images without the use of sedation.

**Methods:**

Utilizing principles of applied behavior analysis (ABA), we developed a protocol for acquiring structural MRI scans in school-aged children with ASD and intellectual impairment. Board certified behavior analysts worked closely with each child and their parent(s), utilizing behavior change techniques such as pairing, shaping, desensitization, and positive reinforcement, through a series of mock scanner visits to prepare the child for the MRI scan. An objective, quantitative assessment of motion artifact in T1- and diffusion-weighted scans was implemented to ensure that high-quality images were acquired.

**Results:**

The sample consisted of 17 children with ASD who are participants in the UC Davis Autism Phenome Project, a longitudinal MRI study aimed at evaluating brain developmental trajectories from early to middle childhood. At the time of their initial scan (2–3.5 years), all 17 children had a diagnosis of ASD and development quotient (DQ) <70. At the time of the current scan (9–13 years), 13 participants continued to have IQs in the range of ID (mean IQ = 54.1, sd = 12.1), and four participants had IQs in the normal range (mean = 102.2, sd = 7.5). The success rate in acquiring T1-weighted images that met quality assurance for acceptable motion artifact was 100 %. The success rate for acquiring high-quality diffusion-weighted images was 94 %.

**Conclusions:**

By using principles of ABA in a research MRI setting, it is feasible to acquire high-quality images in school-aged children with ASD and intellectual impairment without the use of sedation. This is especially critical to ensure that ongoing longitudinal studies of brain development can extend from infancy and early childhood into middle childhood in children with ASD at all levels of functioning, including those with comorbid ID.

**Electronic supplementary material:**

The online version of this article (doi:10.1186/s11689-016-9154-9) contains supplementary material, which is available to authorized users.

## Background

Magnetic resonance imaging (MRI) has played a prominent role in the quest to understand the neurobiological underpinnings of autism spectrum disorder (ASD). MRI is a safe and noninvasive technology that can provide insight into the altered trajectories of brain development that are associated with ASD. In particular, MRI studies hold the promise of longitudinal evaluation of large sample sizes, which is necessary for studies aimed at exploring the heterogeneity of ASD [1, 2]. One limitation to the existing structural MRI literature in ASD, however, is that the majority of studies have focused on older individuals with IQs in the normal range or above. Recent estimates suggest that only about half of the individuals with ASD have IQs in the normal range [3]. Approximately 50–70 % of individuals with ASD have mild to profound comorbid intellectual disability (ID) [3, 4], and we currently have very little information on the structural brain alterations in these more severely affected individuals. This situation is unfortunate because a better appreciation of the neural alterations in more severely affected individuals may inform both our understanding of mechanisms and lead to more individualized interventions.

While there have been several studies focusing on individuals with ASD and comorbid ID [5–11], the majority of these studies utilized sedation or general anesthesia to obtain high-quality images. Although sedation is widely used in clinical practice and is considered safe for use in children with ASD [12], there is minimally increased risk [13], and parents are often hesitant to expose their children to anesthesia when it is not medically indicated. This potentially reduces the sample of individuals with ASD and comorbid ID that are willing to participate in imaging research studies. Since 2006, we have conducted multidisciplinary longitudinal studies of preschool-aged children with ASD and age-matched controls in the Autism Phenome Project (APP). In order to encourage longitudinal participation in the MRI studies, we developed effective techniques for obtaining high-quality MRIs of children as young as 2 years of age during natural nocturnal sleep [14]. The nearly 300 participants in the APP have a broad range of ASD severity and cognitive function (mean Autism Diagnostic Observation Schedule (ADOS) severity score = 8.0, sd = 1.7, range = 4–10; mean DQ = 63.1, sd = 21.5, range = 23.9–136.5). At the time of study entry at 2–3.5 years of age, 68 % had IQs in the range of intellectual disability (DQ <70). Longitudinal evaluations at 4–6 years of age indicate that 38 % of children continued to have IQs in the range of intellectual disability. In order to continue analyses of brain growth trajectories of these children as they enter middle childhood, and due to the difficulty of obtaining MRIs during natural nocturnal sleep at this age, we needed to develop techniques to enable collection of high-quality scans from severely affected, minimally verbal children with ASD, and comorbid intellectual disability.

In this report, we present a summary of methods, based on principles of applied behavior analysis (ABA), for acquiring high-quality structural MRI images in 9- to 13-year-old children with ASD and intellectual impairment without the use of sedation or anesthesia. ABA has been utilized effectively to prepare children for clinical visits to dentists and other medical appointments [15–17], but has not yet been systematically applied to a research MRI setting. We utilized ABA methods of preference assessments, a task analysis, antecedent interventions, and reinforcement strategies in a mock MRI setting to prepare children to successfully complete the MRI scan without sedation. We also implemented a quantitative quality assurance protocol to assess motion artifact in structural T1-weighted and diffusion-weighted images.

Given that we have exceeded our most optimistic expectations and obtained high-quality images with minimal motion artifact from all children imaged thus far, we thought it would be useful to the research community to share the methods that we utilized to acquire these scans. Our goal is to share improved and safer methods for obtaining high-quality images in a broader spectrum of children with ASD. We believe these methods will be generalizable to other neurodevelopmental disorders as well.

## Methods

### Participants

Children were enrolled in the UC Davis MIND Institute Autism Phenome Project (APP). The APP is a longitudinal study, and initial enrollment took place when the children were between 2 and 3.5 years of age. At the time of study entry, diagnostic confirmation was carried out using the Autism Diagnostic Observation Schedule–Generic (ADOS-G) [18, 19] and the Autism Diagnostic Interview–Revised (ADI-R) [20], and developmental ability was obtained using the Mullen Scales of Early Development (MSEL) [21].

Participants for the current study (*n* = 17) were selected based on having ASD and MSEL overall development quotient (DQ) score lower than 70 at study entry and current age between 9 and 13 years. A total of 21 past participants were contacted for the current study. One participant declined to return to attempt another MRI scan, and three participants did not respond to our attempts to contact them. The only exclusion criteria was any contraindications to MRI (e.g., braces). Children were not excluded if they had minimal expressive language ability or severe problem behaviors (e.g., self-injurious behavior). Updated cognitive assessments and diagnostic confirmation using the Differential Ability Scales-II (DAS-II) [22] and ADOS-2 [23] were obtained upon completion of the current MRI scan. All participants and the accompanying parent(s) were screened for any contraindications to MRI. This study was approved by the UC Davis Institutional Review Board and informed consent was obtained from the parents of each child. A letter of assent was provided to children over the age of 11.

### General overview

A board certified behavior analyst (MM or AMS) worked closely with the parents prior to the first mock MRI session to develop a plan that would prepare each child for the procedure based on his/her understanding and use of communication, developmental abilities, and level of anxiety or other comorbid symptoms. At the mock MRI session(s), the behavior analyst implemented a task analysis to break down the complex behavior of lying still during an MRI scan into discrete, teachable steps for the child to learn. Several antecedent-based intervention strategies (i.e., changes to the environment to encourage cooperation with the task) were utilized to familiarize the child with the MRI scanning procedures. If necessary, multiple mock MRI sessions were conducted until the child was able to lie still in the mock MRI scanner with headphones on for 5–10 min. Once this criterion was met, the child was transitioned to the 3T MRI suite for the actual MRI scan. The research staff included the behavior analyst, a research assistant, and a scanner operator; the same individuals were present at all mock and MRI sessions to establish and maintain consistent rapport with each child. An objective quantitative quality assurance protocol to assess for motion artifact in structural T1-weighted and diffusion-weighted images was also implemented.

### Pre-visit preparation

#### Structured interview

The behavior analyst conducted a structured interview with the parents to evaluate their child’s current skill level and ability to follow instructions, particularly from a new person, and to communicate any challenging behaviors that may be elicited from the MRI environment or procedures. A preference assessment was also conducted in order to identify potential reinforcers or preferred materials and activities that could be used with the child during the mock and MRI sessions. Detailed questions are provided in Table 1.

**Table 1:** Pre-visit structured interview questions

Preference Assessment
What are some of your child’s most preferred foods and activities?
Does your child enjoy watching movies or video clips on YouTube?
What are some of his/her favorite movies or things to watch?
Are there any activities/apps/food we should avoid?
What are things that make your child feel happy or comforted?
Are there candies or special treats your child enjoys?
What are things that make your child feel upset?
Does your child have an ipad/tablet they are already familiar with that they could bring to the training sessions?
Would your child find seeing images of his/her brain exciting?
Will a gift card be motivating for your child?
General Compliance
Is your child sensitive to sound?
Does your child tolerate wearing earbuds and/or headphones?
Is your child able to lie still? If so, for how long?
Does your child follow one-step instruction?
Do you think your child would have a preference to have you and/or the behavior analyst in the scanning room with him/her?
Is he/she enrolled in mainstream schooling?
Is he/she enrolled in special education classes? Does your child have an aid?
MRI Safety
Does your child have braces or have any recent dental work done?
Any recent surgeries or metal implants?
Does he/she wear glasses?

#### Video model

Parents were also given the option of showing their child a simple video model of the MRI procedures developed for this study. Narrated in a child-friendly manner, the video features “Spiderman” going through the familiarization protocol in the mock scanner environment and the 3T MRI suite (https://vimeo.com/63857712). A visual storyboard with scenes from the video [see Additional file 1] was also offered to families prior to the mock scanner visit so that the parents could explain the steps involved picture-by-picture with their child. The child-directed script from the video model and a parent-directed handout describing the mock and MRI process are included as Additional files 2 and 3.

### Set-up of pediatric imaging environment

**Fig. 1:**
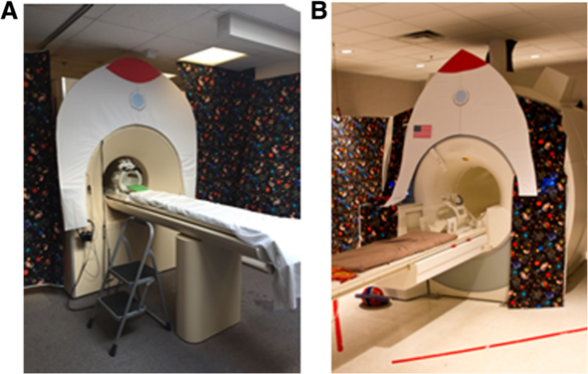
A pediatric-friendly space theme environment for **a** mock MRI scanner and **b** 3T MRI scanner

All mock and MRI sessions were carried out at the UC Davis Imaging Research Center. Both mock and MRI rooms were decorated with an identical child-friendly space theme (Fig. 1).

#### Mock scanner room

The mock scanner (Psychology Software Tools, Inc.) is a wooden mock-up of an MRI system equipped with 6-ft tapered bore, motorized MRI bed, head coil, head stabilizer system, and visual and auditory presentation systems. Two speakers placed in the bore delivered recorded gradient sounds, and a monitor at the end of the bore was used to display visual stimuli (videos, visual timer). Auditory stimuli were delivered through headphones, which were equipped with a computer-assisted potentiometer mounted onto the headband for monitoring head movement with an accuracy of 0.5 mm. In general, only one behavior analyst and one other member of the research team interacted with the child and parents during the mock visit(s). An additional research team member remained in the mock room to control the video/music, pre-recorded gradient sounds, and motion sensor potentiometer; however, this person remained hidden behind a curtain for the duration of each visit. Pediatric silicone or foam earplugs were available for use depending on the child’s preference. A weighted blanket was also available to help decrease the child’s movement and increase comfort.

#### 3T MRI suite

In the 3T MRI suite, visual stimuli (videos, visual timer) were projected onto a screen from a computer in the control room, and audio (music or movie) was presented through pneumatic headphones. A camera mounted on the outside of the bore provided the scanner operator in the control room with a view of the participant lying inside the bore. Because participants in this study were unable to utilize traditional mechanisms to communicate with research staff during the scan (e.g., using the squeeze ball or verbal communication), at least one research team member remained in the MRI room with the child for the duration of the imaging session. An identical weighted blanket to the one in the mock scanner room was available for use in the 3T MRI room.

### Conducting the mock MRI sessions

#### Task analysis

A task analysis was developed to break down the complex behavior of lying still in the mock/MRI scanner into discrete teachable steps involving: (1) entering the mock MRI room, (2) approaching the mock MRI bed, (3) sitting down on the scanner bed, (4) putting on earplugs and/or headphones, (5) lying down on the scanner bed, (6) lowering the head coil, (7) tolerating movement of bed into scanner, (8) tolerating gradient noises, (9) staying still (see the “Motion training” section below), and (10) gradually increasing the amount of time to lie still to 5–10 min. Throughout the task analysis, the child’s preferred video continued to play on the screen in the bore of the mock scanner, which served as a reinforcer for completion of each step. Reinforcers that would naturally encourage the child’s participation were used as often as possible. For example, for steps 4 through 6 of the protocol, naturally occurring reinforcement included putting on the headphones (step 4) in order to hear the preferred video and lying down and lowering the head coil (steps 5 and 6) in order to see the preferred video through a mirror on the head coil.

#### Behavior strategies

To master the steps of the task analysis, a number of antecedent interventions were employed. For all children, the process of shaping was utilized (i.e., gradually reinforcing closer and closer approximations of the desired behavior). A brief description of the various strategies utilized and specific examples from mock MRI sessions are provided below.

- *Pairing* is the process of presenting highly preferred materials in an otherwise neutral or non-preferred environment, which over time leads to the environment itself becoming highly reinforcing. In the mock MRI room, this occurred by having a number of highly preferred items on hand to assist the participant with feeling comfortable with the behavior analyst and the environment. For some children who exhibited significant resistance to being at the Imaging Research Center, a gradual desensitization approach was utilized, whereby the only expectation for the first mock MRI visit was to enter the mock MRI room before slowly adding more steps for the child to complete. Participants were always allowed as much time as they needed and offered reinforcement before advancing to the next step, thus ensuring the visits and overall experience always ended on a positive a note.
- *Choices* Giving the participant a variety of choices served to increase motivation and give the child some degree of control. For example, the child could choose which video to watch or other reinforcers they wanted during the mock MRI session.
- *Premack principle* Using “first/then” instructions provided participants with a verbal understanding of both expectations and the schedule. The behavior analyst would give the instruction (“first keep your hands down”) followed by the reinforcer (“then we will start the movie”).
- *Behavior momentum* The strategy involved asking the child to do several, relatively easy tasks or behaviors in rapid succession so that the child was then more capable and likely to complete the desired behavior (e.g., “Give me a high five!”; “Give me a low five!”; “OK, now lie down”; “Great job!”).
- *Peer modeling* A sibling and/or parent modeled steps 2–7 of the task analysis—from approaching and sitting on the mock scanner bed, to lying down on the bed, and moving into the bore of the scanner—in order to aid the child with understanding the process. Parents or siblings would express their positive experience and model calm behavior.
- *Visual storyboards* As part of the pre-visit procedures, parents received a storyboard that they could share with their child, which explained with simple words and pictures what would happen at the visit. Some parents brought the story with them to the mock MRI sessions to show their child pictures of each step.
- *Visual timer* A visual countdown timer (http://www.online-stopwatch.com/countdown-clock/) was projected onto the screen below the video, allowing the child to monitor how much time remained for each MRI sequence.
- *Verbal countdowns* For some participants, the task of remaining still in the bore of the mock MRI scanner was too difficult, so lying still was practiced beforehand and outside of the bore. The behavior analyst provided a verbal countdown (5, 4, 3, 2, 1!) to assist the child in their understanding of how long to expect to remain still. The countdown generally began with a very minimal demand, (e.g. 5 s) and slowly increased in duration. Countdowns were always paired with verbal praise, usually during and after completing the stated length of time. Usually, the behavior analyst specified the length of time; however, on some occasions, the child was given the choice of the length of time they thought they could achieve.
- *Verbal reminders* Some participants responded well to verbal reminders to lie still. Verbal reminders were often used in conjunction with the visual countdown timer. The behavior analyst gave verbal feedback and praise to the child as the time decreased (e.g., “Only 2 minutes left, you are doing great, keep lying still!”).

#### Motion training

The motion potentiometer mounted onto the headphones was utilized for teaching participants to remain still while lying inside the mock scanner, watching their preferred video, and listening to the audio through headphones. A response-cost procedure, in which the contingent loss of a reinforcer (video) produces a decrease in the frequency of behavior (motion), was implemented to teach the child to lie still. If the child exceeded a pre-set movement criterion, the video was automatically interrupted (i.e., the screen blacked out) to provide both feedback and a contingent consequence for moving too much. The video resumed when the child’s motion fell below the movement criterion. The movement criterion was adjusted according to the child’s current ability and was gradually made more stringent as the child became more successful. The initial movement criterion was set to 5 mm and gradually decreased to 1 mm. The gradient noises for the structural and diffusion-weighted scans were played at increasingly louder volumes during motion training.

#### Criterion for completion of mock training

The criterion to complete the mock scan training was being able to lie still (<1 mm) in the mock scanner while tolerating the gradient noises at full volume for 5–10 min. Each mock MRI session lasted less than 2 h. Children were given a second mock MRI session, if needed, to learn the procedure and to reach the criterion. Once the criterion was reached, the child was transitioned to the 3T MRI suite, either on the same day or in a subsequent visit.

### MRI session

Participants and their parents were screened for MRI safety, and all personal items were removed and placed in lockers outside the control room. Parents were encouraged to have children dress in cotton clothing with no pockets (e.g., sweatpants) to minimize the screening time. Upon entry into the MRI room, all steps previously mastered from the task analysis in the mock room were repeated. The child was again supported through each step and provided with either social or tangible reinforcement upon successful completion of each step. Training in the MRI moved quickly due to the child’s previous success during the mock training.

Scans were acquired with a 3T Siemens TIM Trio whole-body MRI system (Siemens Medical Solutions, Erlangen, Germany) using an 8-channel head coil. Sequences were collected in the following prioritized order: (1) sagittal T1 localizer; 0:09, (2) 3D T1-weighted MPRAGE (TR 2170 ms; TE 3.5 ms; FOV 256 mm; FA 7; 192 slices, 1.0 mm slice; 5:10), and (3) diffusion-weighted scan (TR 11500 ms; TE 91 ms; FOV 243 mm; 72 axial slices, 1.9 mm isotropic; 30 diffusion directions; 5:45).

The visual timer was projected on the screen below the video to provide the time remaining in each sequence. The timer was set to the acquisition time for the T1 localizer and MPRAGE sequences and synced to the start of each sequence to provide the participant precise feedback on how much longer they needed to lie still. Upon completion of the MPRAGE, the timer was reset for the duration of the diffusion-weighted sequence and synced to the initiation of the scan. During each sequence, the behavior analyst monitored the child’s movement and provided feedback to the participant. Depending on each child’s needs, the behavior analyst either remained in the scanner room, where direct physical contact was possible (e.g., squeezing leg) or in the control room, where verbal communication was made through a microphone connected to the child’s headphones. The motion potentiometer used in the mock MRI scanner was not available in the MRI room; in order to mimic the response-cost procedure training carried out in the mock MRI sessions, the video projection was manually blacked out when the child moved excessively to provide instant feedback to the child about their movement. The parent had the option of either remaining with the child in the MRI room or in the control room, where he/she was able to verbally communicate with the child through the headphones. Sequences were repeated as necessary until objective quality assurances for minimal motion artifact were met. If necessary, children were allowed to get up to take a break between sequences.

### Quantitative assessment of motion artifact

**Fig. 2:**
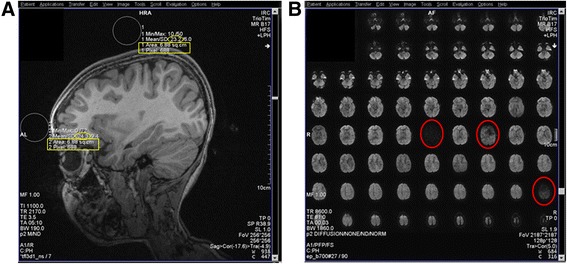
Objective motion quality assurance protocol. **a** Depicts ROI placement for T1-weighted assessment. The size of the ROI was between 650 and 850 pixels or 6.50 and 8.50 cm^2^ (*yellow rectangle*). The ratio of the mean signal intensity (*yellow ovals*) anterior/superior ROIs was required to be ≤2.0. **b** Depicts an example of slice dropout (*red circles*) in one volume of a diffusion-weighted scan. Volumes were excluded if any slice dropout was present, and the number of excluded volumes was required to be ≤5 out of a total of 30 volumes

We utilized procedures developed by the Pediatric Imaging, Neurocognition, and Genetics (PING) study (http://pingstudy.ucsd.edu), a multisite study of individuals aged 3 to 21 years, to objectively quantify motion in T1- and diffusion-weighted scans. The PING motion protocol has been validated to produce high-quality MRI images [24, 25]. The quality assurance (QA) procedure for the T1-weighted scans measured the average signal intensity from two circular regions of interest (ROIs) with an area between 6.5 and 8.5 cm^2^ (650–850 2D pixels) in size. ROIs were drawn in the sagittal view at the level of the orbit; one superior to the skull and the other anterior to the orbit (Fig. 2a). The ratio of mean signal intensity (mean anterior/mean posterior) was required to be ≤2.0 in order to accept the quality of the image. For diffusion-weighted images, the number of volumes that needed exclusion because of slice dropout was recorded and was required to be ≤5 (out of 30 possible volumes) to meet the QA threshold (Fig. 2b).

## Results

### Participant characteristics

The sample consisted of 17 children with ASD (mean age = 11.0 years; sd = 1.4; range = 9.0–13.3), selected based on having DQ scores <70 at the time of study entry (age 2–3 years). At study entry, mean DQ was 51.7 (sd = 11.3; range = 36.2–68.8). At the time of the current MRI scan, mean IQ was 67.8 (sd = 24.2, range = 41.1–108.3). Of the 17 participants, 13 still had IQs in the range of ID (mean IQ = 54.1, sd = 12.1), and the remaining four had IQ scores that had moved into the normal range (mean = 102.2, sd = 7.5). The mean ADOS severity score at the time of the current study was 7.4 (sd = 2.1). Individual scores are provided in Table 2. Individual *t* scores and age-equivalent scores for DAS subscales are presented in [see Additional file 4: Table S1].

**Table 2:** Individual participant scores and mock and MRI session data

Participant	Age (years)	IQ	VQ	NVQ	ADOS module	ADOS total score	# mock sessions	# MRI sessions	# T1 attempts (QA ratio)	# DTI attempts (# vol excluded)
1	9.0	67.8	63.3	72.3	2	18	2	1	1 (1.06)	1 (0)
2	9.4	41.3	40.0	42.6	1	23	2	1	1 (1.05)	1 (0)
3	9.7	73.9	66.3	81.6	3	6	2	1	2 (1.30)	1 (0)
4	9.7	41.1	40.0	42.3	1	19	2	1	5 (1.48)	1 (4)
5	9.9	47.7	40.0	55.4	1	19	1	1	5 (1.11)	N/A
6	10.0	49.8	40.0	59.5	1	17	2	1	3 (1.16)	1 (1)
7	10.1	52.2	40.0	64.4	1	17	1	1	2 (1.55)	1 (0)
8	11.3	49.3	38.2	60.3	1	22	2	1	2 (1.30)	2 (2)
9	11.9	78.3	78.3	78.3	3	8	1	1	1 (0.77)	1 (0)
10	12.3	52.8	48.3	57.3	3	16	2	1	2 (1.33)	2 (0)
11	12.3	43.0	26.7	59.3	1	21	2	1	3 (1.46)	1 (3)
12	12.9	47.5	40.0	55.0	1	24	1	1	3 (1.02)	1 (0)
13	13.2	57.4	40.0	74.9	2	24	2	1	1 (1.04)	1 (0)
14	10.2	108.3	98.5	118.0	3	24	1	1	2 (1.06)	1 (0)
15	11.3	107.1	118.8	95.5	3	16	1	1	1 (0.91)	1 (0)
16	11.3	101.5	101.5	101.5	3	13	1	1	1 (0.88)	1 (0)
17	13.3	91.8	72.3	111.3	3	9	2	1	1 (1.05)	1 (0)

IQ—DAS General Conceptual Ability standard score; VQ—DAS verbal standard score; NVQ—DAS nonverbal standard score; ADOS total score—social affect + restricted and repetitive behavior

### Scanning success rate

**Fig. 3:**
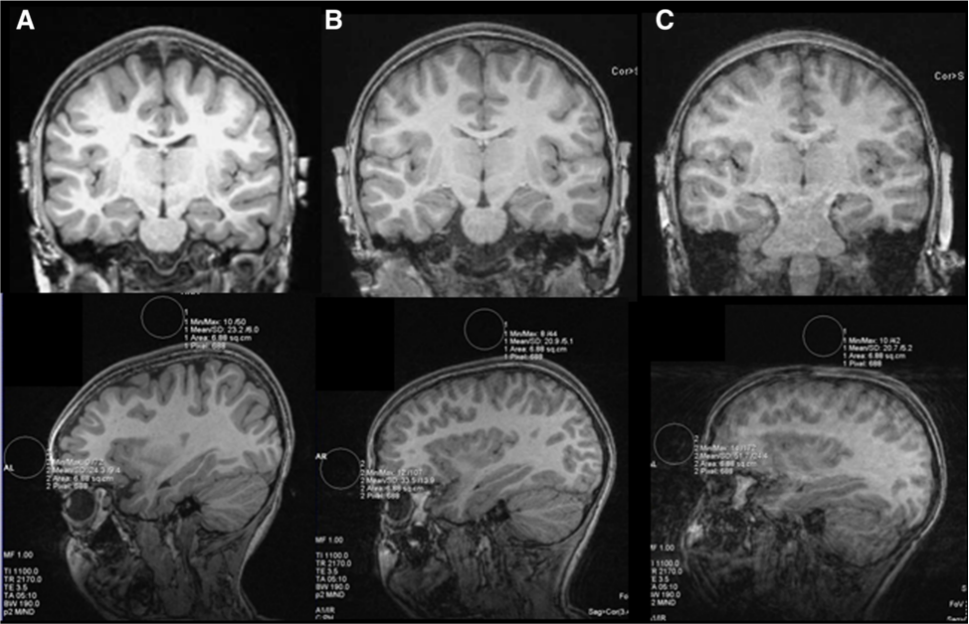
Examples of T1-weighted images with varying degrees of motion. Coronal slices of the scans (*top*) and sagittal slices depicting the motion QA assessment for **a** a scan from with minimal motion (QA ratio = 1.05; participant 2 in Tables 1 and 2), **b** a scan with moderate motion (QA ratio = 1.48; participant 4 in Tables 1 and 2), and **c** an unsuccessful MPRAGE attempt for the same participant depicted in **b** demonstrating an unacceptable level of motion (QA ratio = 2.5)

T1-weighted images that exceeded QA standards were acquired in all 17 participants. For all participants, only one MRI visit was necessary to acquire high-quality scans. Within the single MRI visit, the number of times the T1-weighted sequence was initiated before acquiring a scan that met QA standards ranged from 1 to 5 attempts (mean = 2.12 attempts sd = 1.32) (see Table 2 for individual data). For the four participants with current IQs in the normal range, an acceptable T1-weighted scan was acquired on the first (75 %) or second attempt (25 %). Of the 13 participants with IQs in the range of ID, 62 % had an acceptable T1-weighted scan after the first (*n* = 4) or second (*n* = 4) attempt of the sequence. Three attempts were required for three (23 %) participants, and for two (15 %) participants, an acceptable T1-weighted scan was acquired on the fifth attempt. If the participant was observed to have excessive movement during the scan, the sequence was stopped early. The mean QA assessment of motion artifact in the T1-weighted images was a ratio of 1.15 (sd = 0.22, range = 0.77–1.55). Individual data on the number of T1-weighted scan attempts and QA ratios of the successful scan are provided in Table 2. Figure 3 depicts scans with low motion (QA ratio = 1.05), moderate motion but still acceptable (QA ratio = 1.48), and a scan that was excluded from the study for exceeding acceptable levels of motion (QA ratio = 2.50).

Diffusion-weighted images that exceeded QA standards were obtained in all four participants with IQs in the normal range on the first attempt. For the 13 participants with IQs in the range of ID, successful images were acquired in 12 participants (92 %) on either on the first (*n* = 10) or second (*n* = 2) attempt. The diffusion-weighted sequence was not attempted in one of the participants who required five attempts at acquiring a successful T1-weighted scan. For QA assessment of diffusion-weighted images, 12 participants (71 %) had 0 volumes with slice dropout and the remaining four participants had a range from 1 to 4 volumes that were excluded for slice dropout. Individual data on the number of DTI attempts and number of volumes excluded for slice dropout are provided in Table 2.

### Mock MRI training

The number of mock training sessions never exceeded into two visits. All four participants with IQs in the normal range required only one mock visit. Of the participants with IQs in the range of ID, four (31 %) required only one mock visit prior to the MRI session, and the remaining nine (69 %) participants had two mock training sessions.

### Behavior strategies used

Table 3 provides detailed information about which antecedent-based intervention strategies were utilized for each participant. All participants benefitted from the process of pairing and being given choices. The visual timer was also beneficial for a majority (82 %) of participants, and the Premack principle (first/then) was utilized in 41 % of participants. Peer modelling and the visual storyboard were effective for 29 % of participants. Verbal countdowns and verbal reminders were used in 18 and 24 % of participants. Narratives for the mock and MRI sessions for two participants (participant 4 and 5) provide detailed examples of how these strategies were used [Additional file 5].

**Table 3:** Frequencies of behavior intervention strategies

Participant	Pairing	Choices	Premack principle (1st/then)	Behavior momentum	Peer model	Visual storyboard	Visual timer	Verbal countdown	Verbal reminders
1	x	x					x		
2	x	x	x		x		x		
3	x	x	x						x
4	x	x	x	x			x		x
5	x	x			x	x	x	x	
6	x	x	x		x	x			
7	x	x					x		x
8	x	x	x			x	x		
9	x	x					x		
10	x	x	x		x	x	x	x	
11	x	x					x		
12	x	x					x	x	
13	x	x			x		x		
14	x	x					x		
15	x	x				x			
16	x	x	x				x		
17	x	x					x		x
Totals (percent)	17 (100 %)	17 (100 %)	7 (41 %)	1 (6 %)	5 (29 %)	5 (29 %)	14 (82 %)	3 (18 %)	4 (24 %)

Participant numbers match those depicted in Table 1. Participants 1–13 have IQs in the range of ID

## Discussion

Children with ASD and comorbid intellectual disability are under-represented in imaging studies of ASD. We sought to determine the feasibility of acquiring T1- and diffusion-weighted images in children with ASD and IQs in the range of ID without the use of sedation. Although we anticipated only partial success in this endeavor, we were able to obtain high-quality scans with minimal motion artifact from all children who participated in this study.

While mock scanning procedures are commonly used for increasing compliance in MRI studies in challenging populations, including children with higher functioning ASD, they have not yet been widely utilized for scanning more severely affected children. We developed a mock scanning protocol based on principles of behavior analysis that involves experienced behavior analysts working closely with the parents and their child. While the strategies implemented here are not new to the field of ABA, they are not frequently used to support children with ASD in research settings. The behavior analysts utilized a number of antecedent-based behavioral strategies to increase compliance among the participants and gradually improve their understanding of the expectations in place during the MRI scan. These strategies were successful even in children with challenging behaviors and minimal expressive language. All children in the study had sufficient receptive language to understand and cooperate with instructions delivered by the behavior analyst. The age equivalent scores on the DAS verbal similarities subscale for children in this study with intellectual impairment ranged from 58 to 88 months (mean 61.7 months).

The protocol described in this study is most successful for children who have preferred videos or video clips and would need to be modified for children who are not motivated by watching videos. For example, for one such child in this study, songs from her favorite music artist were played instead of a video. Although this protocol was developed for children with ASD and intellectual impairment, we believe that some of the behavior change strategies (e.g., visual countdown timer) would be useful even in higher functioning individuals with ASD to minimize motion artifact and improve the quality of imaging data. Recent evidence suggests that even small amounts of head motion can lead to spurious results in imaging data [26, 27].

## Conclusions

Our experiences make us confident that acquiring high-quality structural MRI scans in severely affected, minimally verbal children with ASD, and comorbid intellectual disability is possible at a high rate of success without the use of sedation. The current MRI literature in ASD is largely limited to studies of high functioning individuals with ASD, who represent only part of the entire spectrum. In order to gain a complete understanding of the neural alterations associated with ASD, we must evaluate individuals at all severity levels. The current perception is that it is too difficult to image school-age children with ASD and comorbid intellectual disability unless they undergo sedation or anesthesia. The goal of the efforts reported here were not only to enable our own longitudinal studies of children at all severity levels but also to encourage the field of neurodevelopmental disorders to re-evaluate the potential of using these safe and effective means of obtaining MRI scans in severely affected children.

## Additional files

Additional file 1: Video story board. (PDF 7870 kb)

Additional file 2: MRI Video Scan Script. (PDF 142 kb)

Additional file 3: MRI Video Scanning—Parent Handout. (PDF 33.6 kb)

Additional file 4: Table S1. *T* scores and age equivalent scores for the DAS subscales. (PDF 96.1 kb)

Additional file 5: Supplementary materials: Individual narratives. (PDF 75 kb)

## References

[1] Lenroot RK, Yeung PK (2013). Heterogeneity within autism spectrum disorders: what have we learned from neuroimaging studies?. Front Hum Neurosci.

[2] Amaral DG, Schumann CM, Nordahl CW (2008). Neuroanatomy of autism. Trends Neurosci.

[3] CDC (2014). Prevalence of autism spectrum disorder among children aged 8 years—autism and developmental disabilities monitoring network, 11 sites, United States, 2010. MMWR Surveill Summ.

[4] Fombonne E (2003). Epidemiological surveys of autism and other pervasive developmental disorders: an update. J Autism Dev Disord.

[5] Erbetta A (2014). Neuroimaging findings in 41 low-functioning children with autism spectrum disorder: a single-center experience. J Child Neurol.

[6] Manes F (1999). An MRI study of the corpus callosum and cerebellum in mentally retarded autistic individuals. J Neuropsychiatry Clin Neurosci.

[7] Riva D (2011). Basal forebrain involvement in low-functioning autistic children: a voxel-based morphometry study. AJNR Am J Neuroradiol.

[8] Zeegers M (2009). No differences in MR-based volumetry between 2- and 7-year-old children with autism spectrum disorder and developmental delay. Brain Dev.

[9] Nordahl CW (2007). Cortical folding abnormalities in autism revealed by surface-based morphometry. J Neurosci.

[10] Schumann CM (2004). The amygdala is enlarged in children but not adolescents with autism; the hippocampus is enlarged at all ages. J Neurosci.

[11] Hazlett HC (2005). Magnetic resonance imaging and head circumference study of brain size in autism: birth through age 2 years. Arch Gen Psychiatry.

[12] Ross AK (2005). Moderate sedation for MRI in young children with autism. Pediatr Radiol.

[13] Rappaport BA (2015). Anesthetic neurotoxicity—clinical implications of animal models. N Engl J Med.

[14] Nordahl CW (2008). Brief report: methods for acquiring structural MRI data in very young children with autism without the use of sedation. J Autism Dev Disord.

[15] Hernandez P, Ikkanda Z (2011). Applied behavior analysis: behavior management of children with autism spectrum disorders in dental environments. J Am Dent Assoc.

[16] Conyers C (2004). An evaluation of in vivo desensitization and video modeling to increase compliance with dental procedures in persons with mental retardation. J Appl Behav Anal.

[17] McComas JJ, Wacker DP, Cooper LJ (1998). Increasing compliance with medical procedures: application of the high-probability request procedure to a toddler. J Appl Behav Anal.

[18] DiLavore PC, Lord C, Rutter M (1995). The pre-linguistic autism diagnostic observation schedule. J Autism Dev Disord.

[19] Lord C (2000). The autism diagnostic observation schedule-generic: a standard measure of social and communication deficits associated with the spectrum of autism. J Autism Dev Disord.

[20] Lord C, Rutter M, Le Couteur A (1994). Autism Diagnostic Interview-Revised: a revised version of a diagnostic interview for caregivers of individuals with possible pervasive developmental disorders. J Autism Dev Disord.

[21] Mullen EM. Mullen Scales of Early Learning (AGS ed.). Circle Pines, MN: American Guidance Service Inc.; 1995.

[22] Elliott CD (2007). Differential ability scales.

[23] Lord C (2012). Autism Diagnostic Observation Schedule.

[24] Fjell AM (2012). Multimodal imaging of the self-regulating developing brain. Proc Natl Acad Sci U S A.

[25] Jernigan TL (2016). The Pediatric Imaging, Neurocognition, and Genetics (PING) data repository. Neuroimage.

[26] Power JD (2012). Spurious but systematic correlations in functional connectivity MRI networks arise from subject motion. Neuroimage.

[27] Yendiki A (2013). Spurious group differences due to head motion in a diffusion MRI study. Neuroimage.

