# Supplementary material for: Methods for acquiring MRI data in children with autism spectrum disorder and intellectual impairment without the use of sedation
Source: J Neurodev Disord. 2016 May 5;8:20. doi: 10.1186/s11689-016-9154-9 (PMC4858915; doi:10.1186/s11689-016-9154-9)

Welcome to the Imaging Research Center!

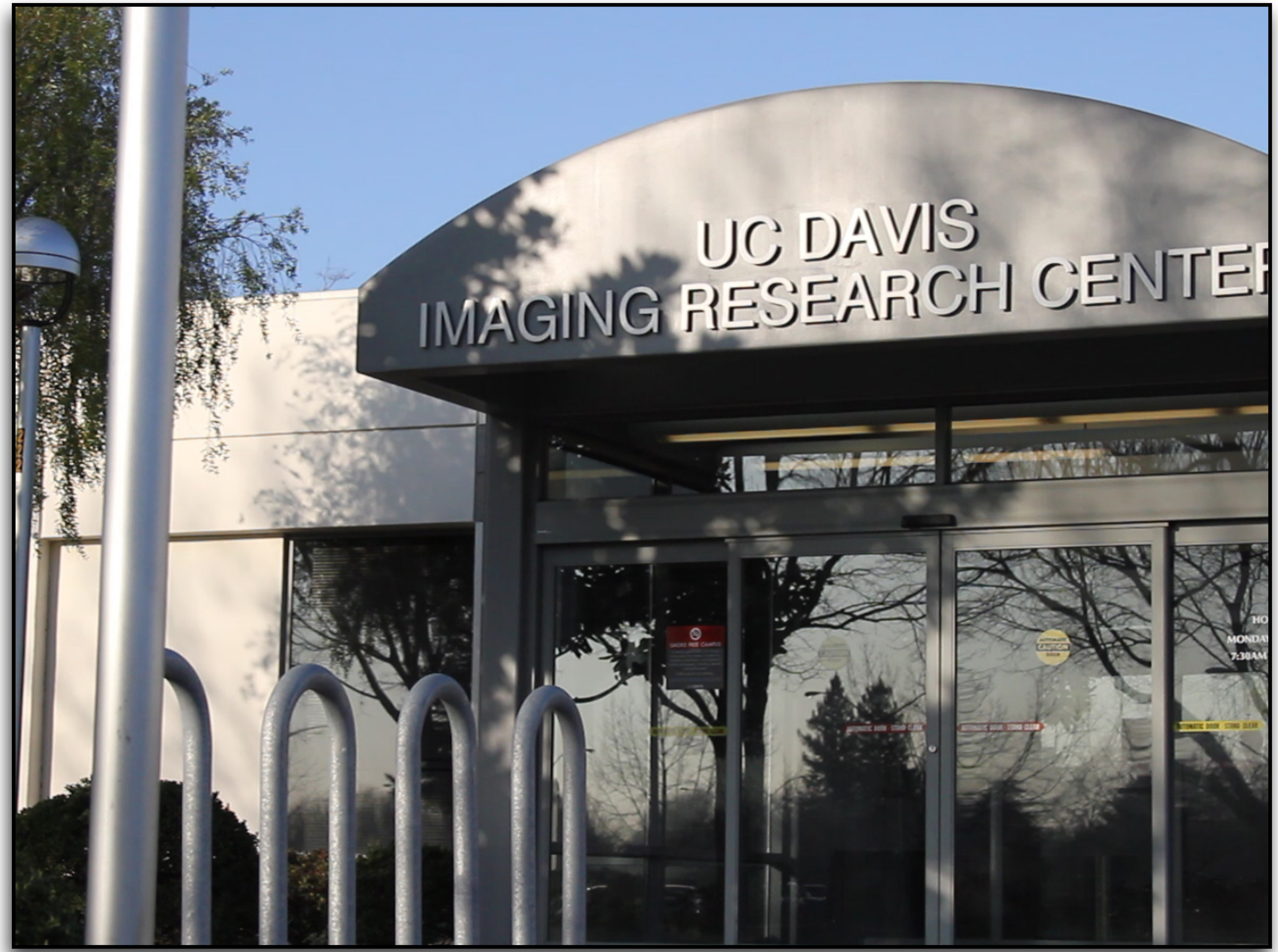

Spider-Man is here to help prepare you for the real MRI space mission where we will take pictures of your brain! To help get you ready for the space mission, you will visit our practice MRI spaceship and do all the things Spider-man does in the pictures.

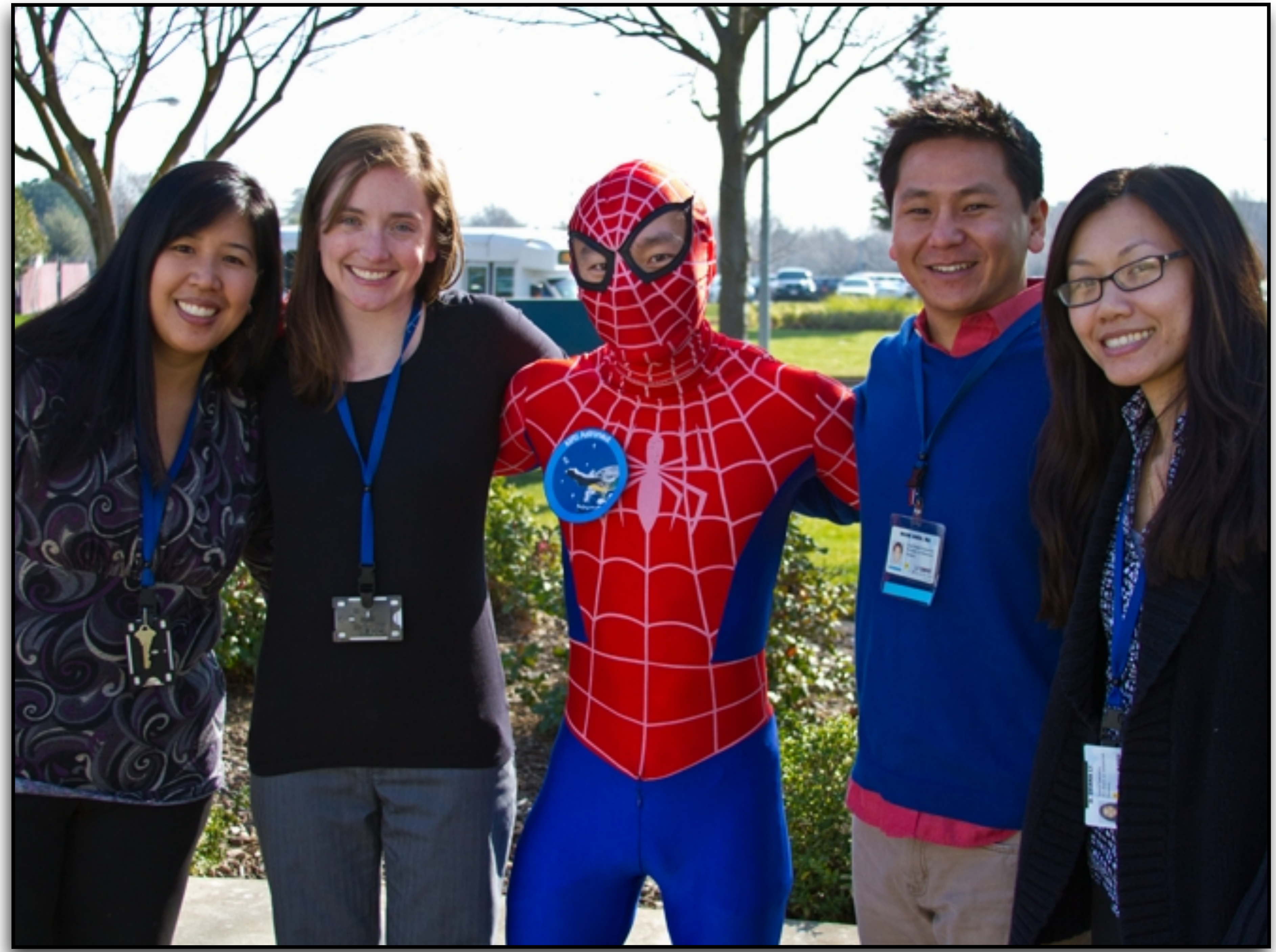

He is really excited because he is taking an iPad with him and will get to take breaks from practicing for the MRI to play games.

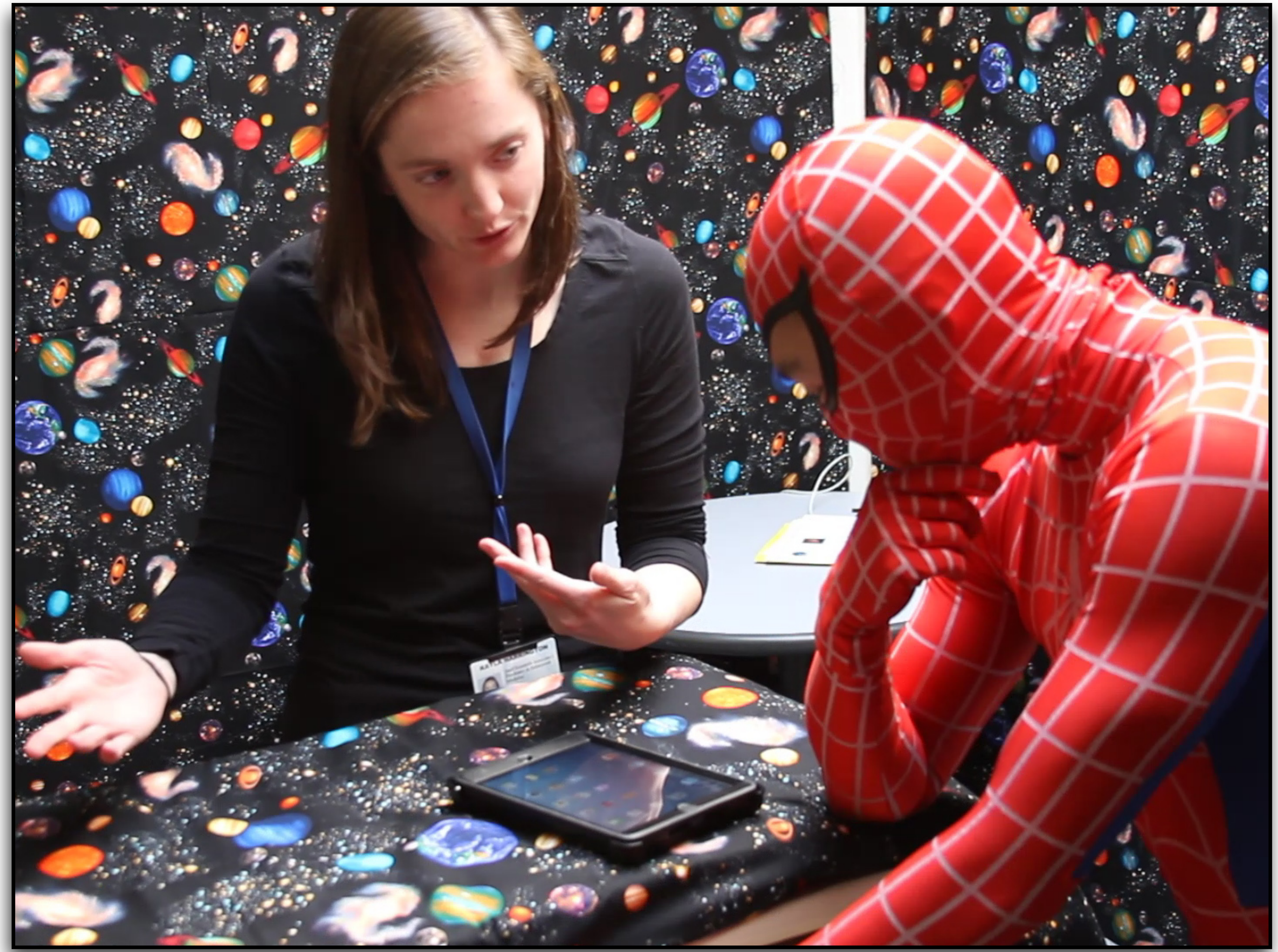

First, Spider-Man sees the room where the pretend spaceship is. There are planets and stars all around! Before he starts practicing, Spider-Man picks a movie to watch for when he is ready to go into the space tunnel.

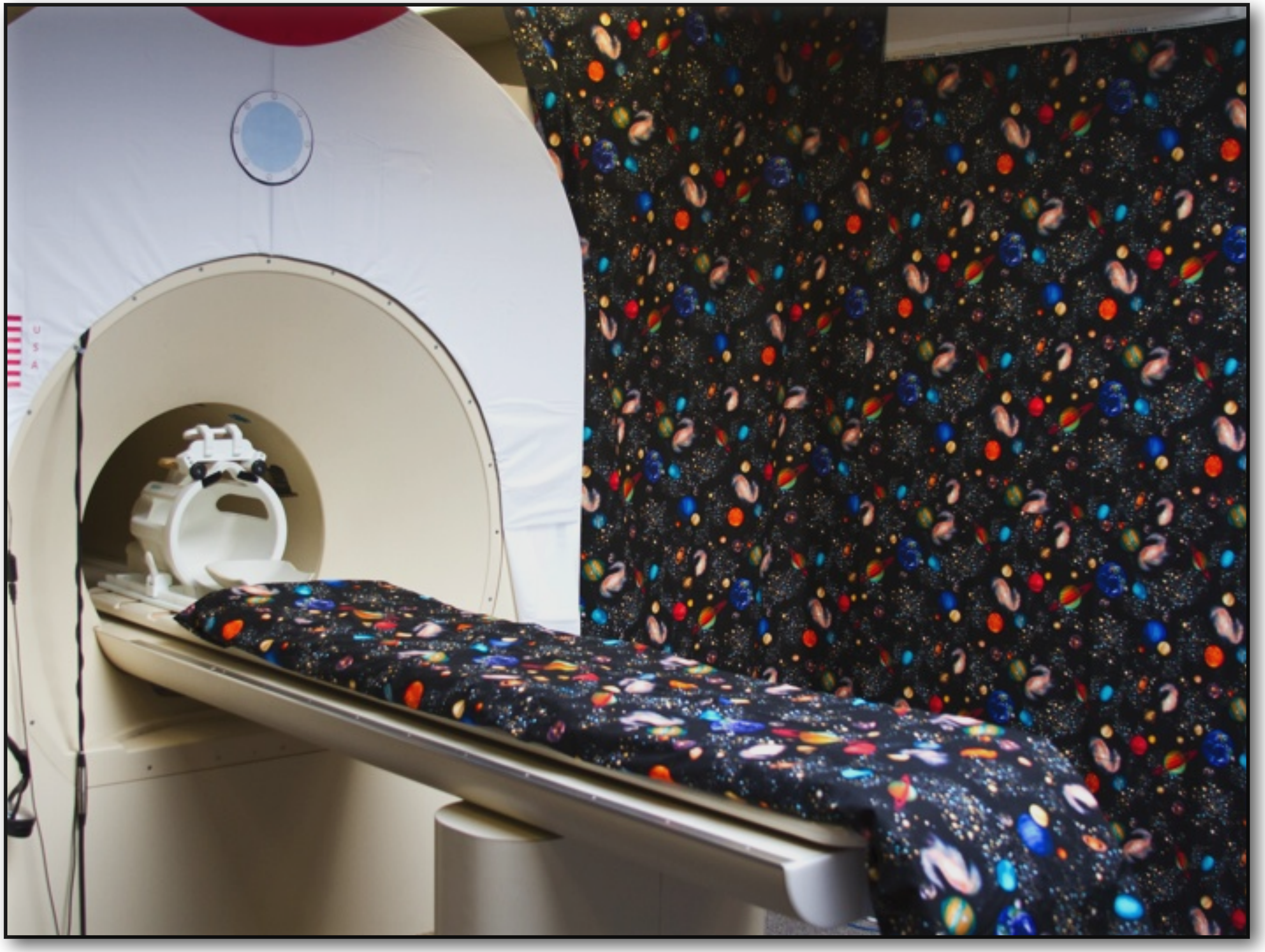

We show Spider-Man how to make the spaceship move so that he can try it too!

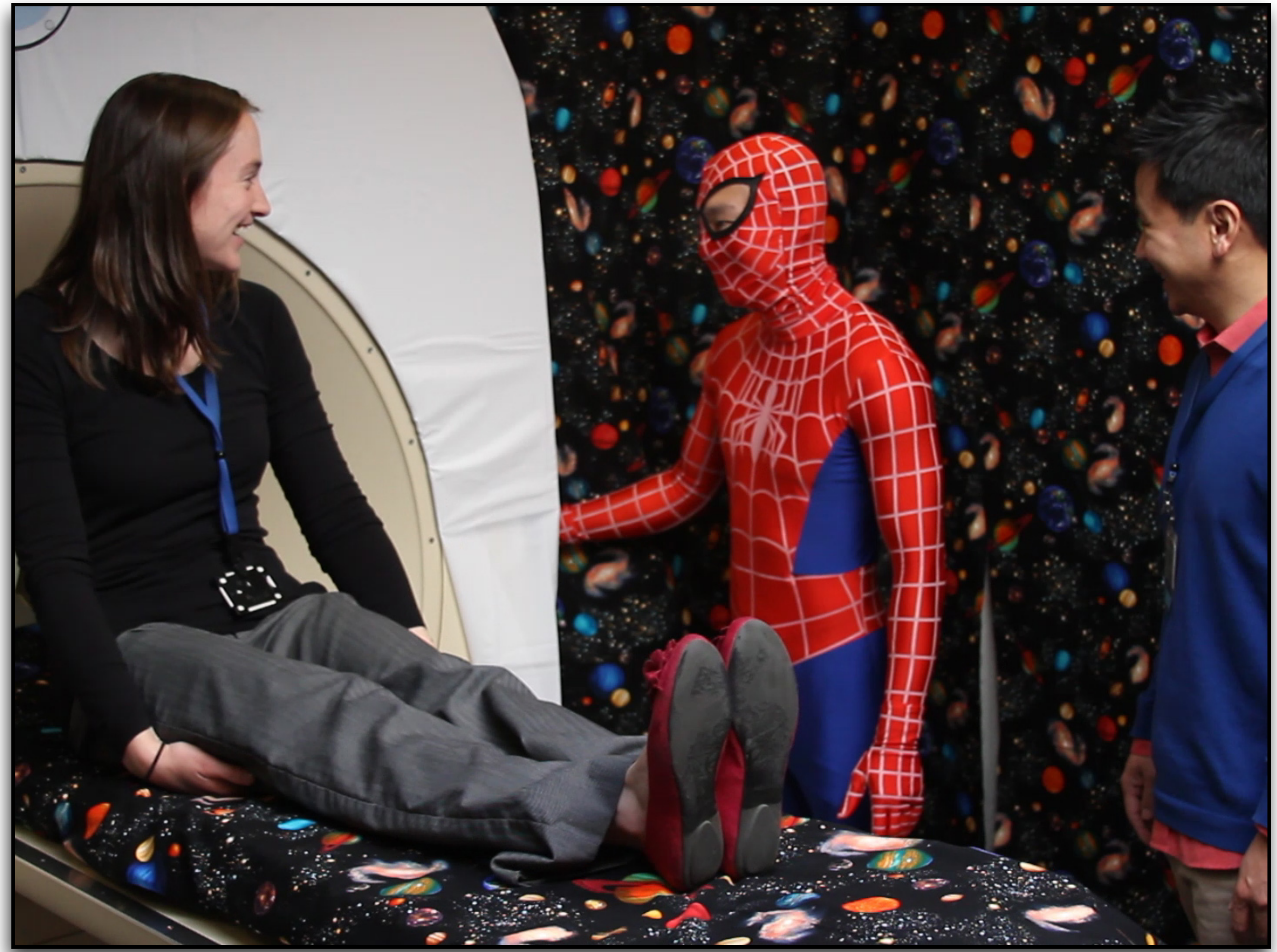

Spider-Man is excited about taking a trip into space, but he needs to climb onto the spaceship first.

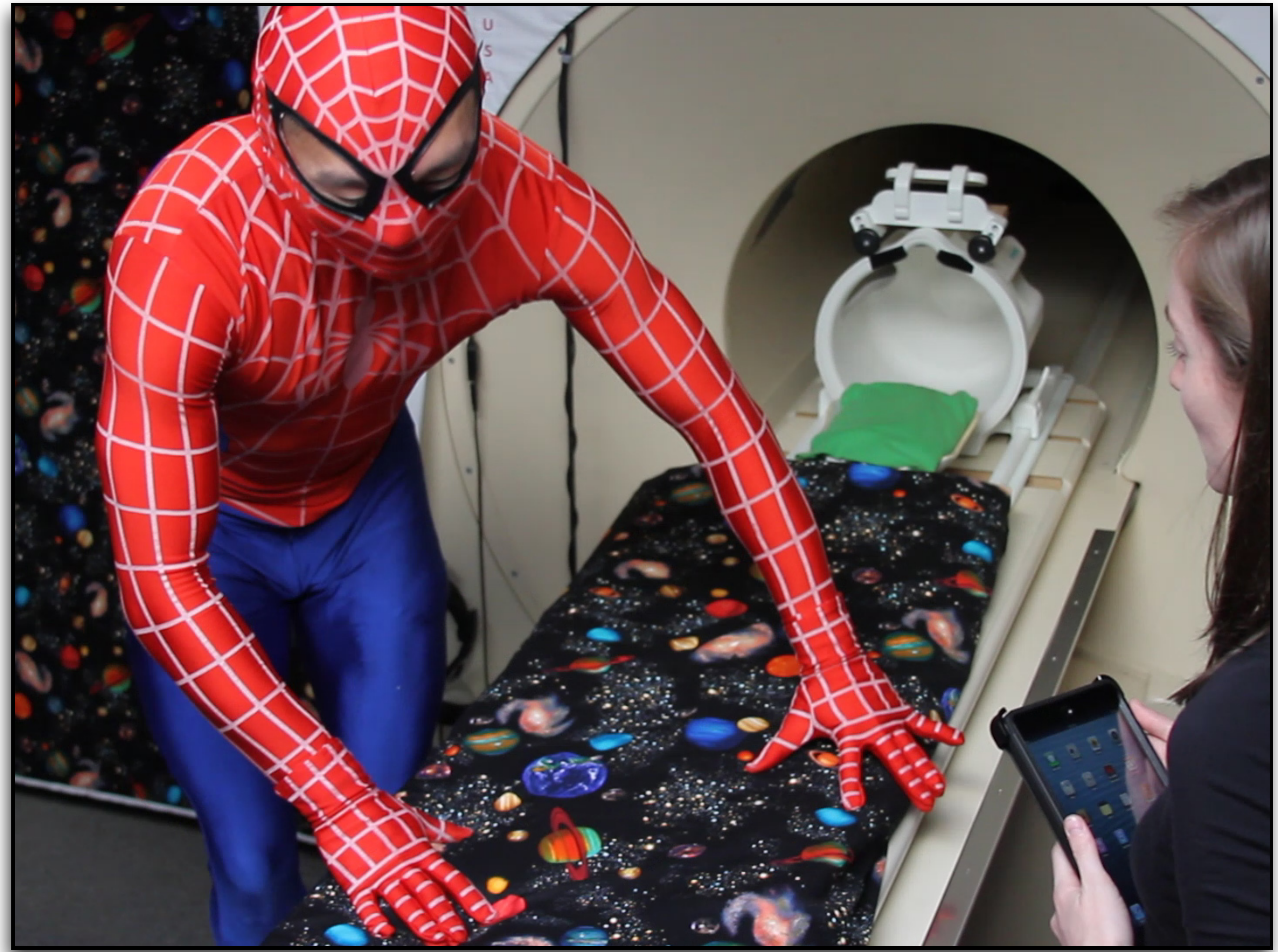

He puts on his special space headphones which help keep track of how much he is moving his head. Remember, the most important part is to stay still!

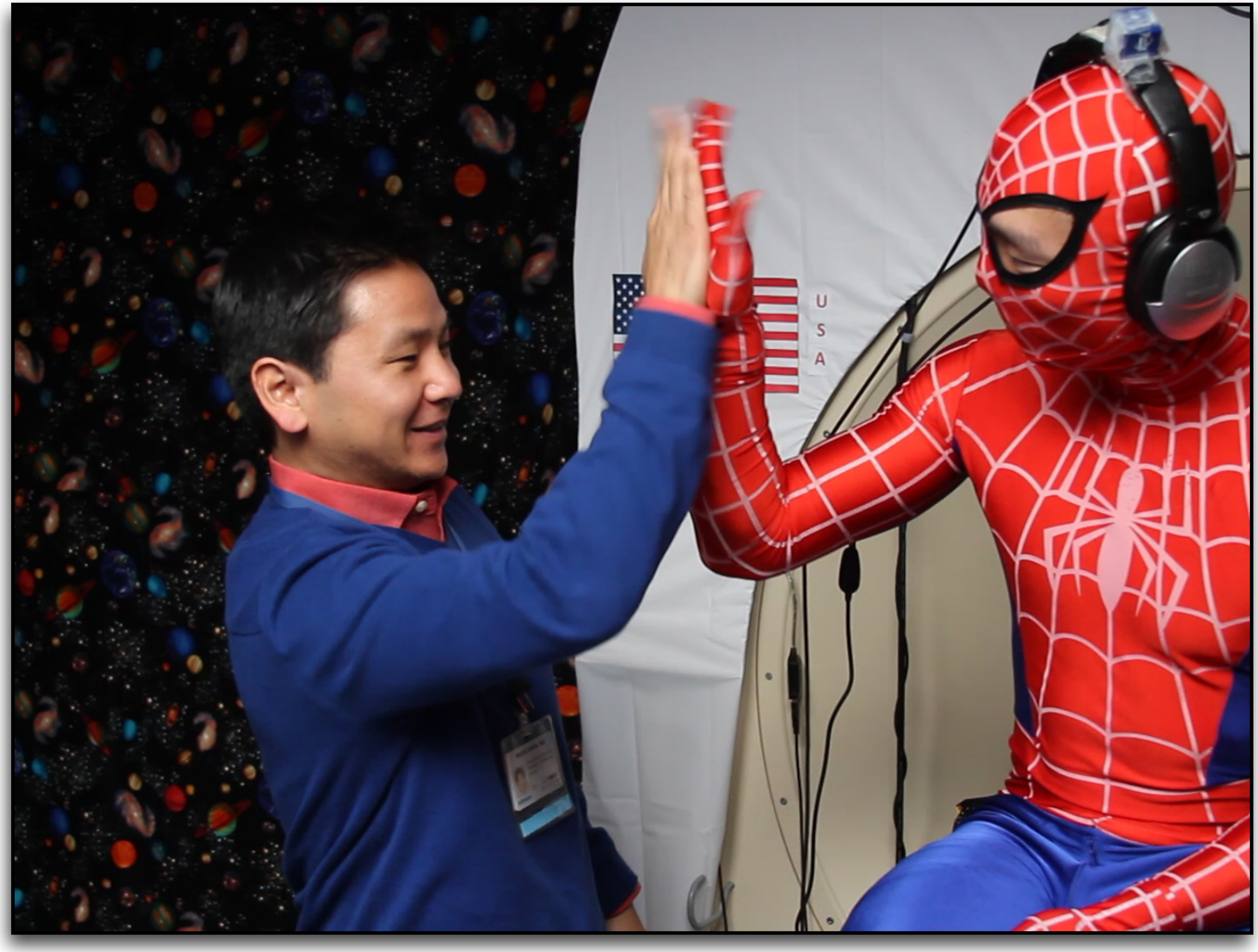

After he has his special headphones on, Spider-Man lies down on the bed.

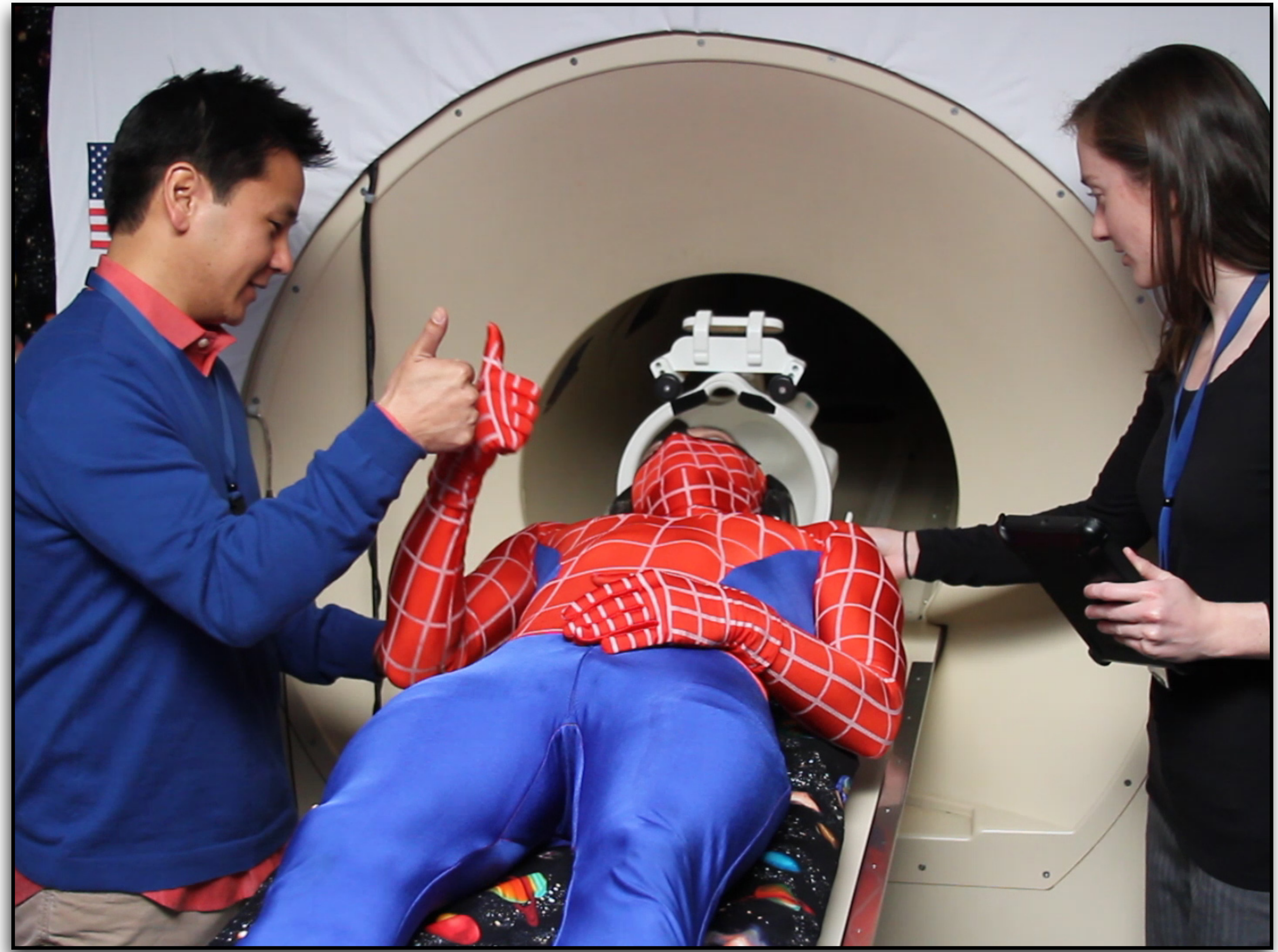

We help Spider-Man put on his space helmet so he can watch the movie he picked out. Now he is ready for take-off!

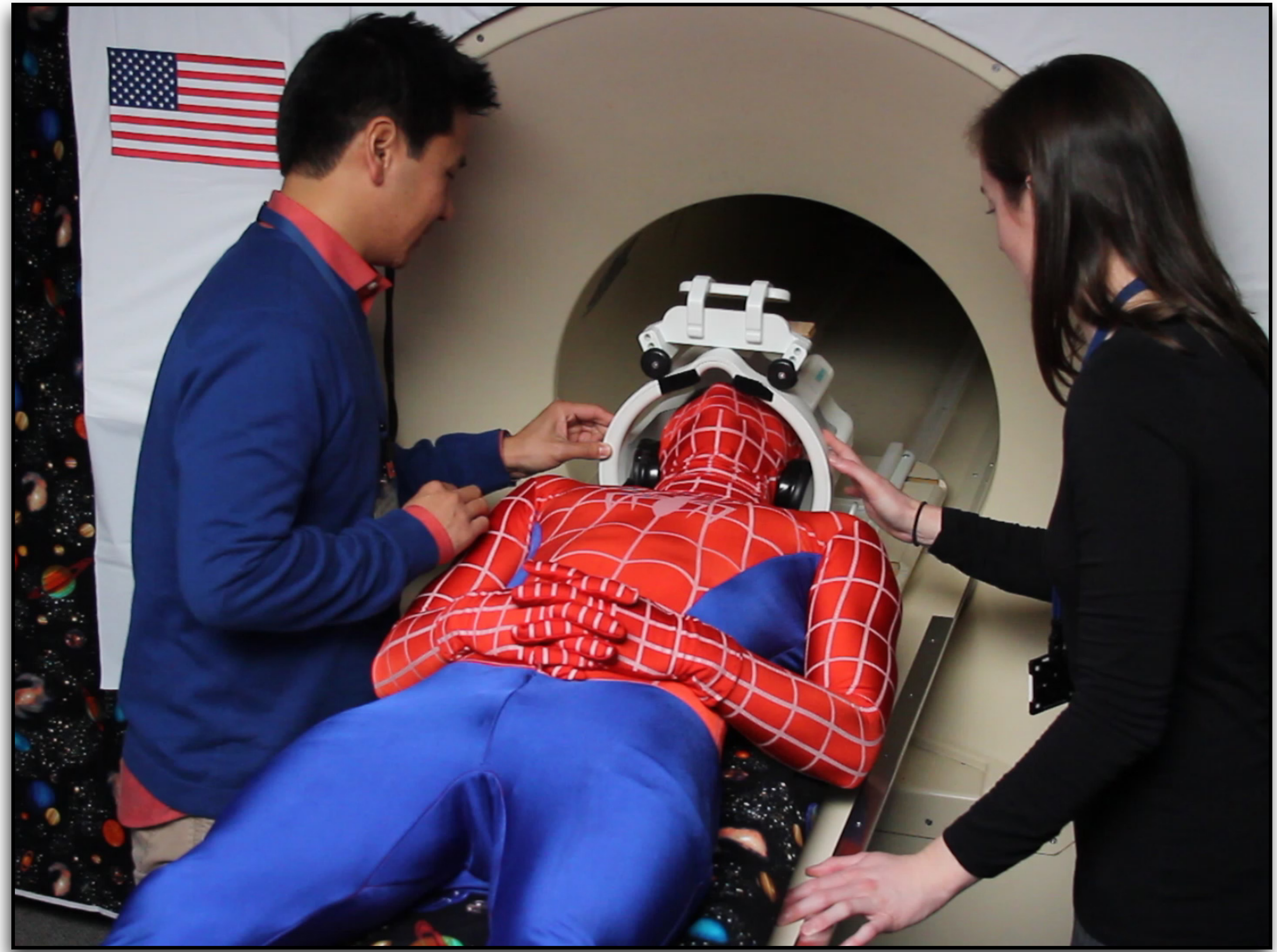

3-2-1 Blast Off!!!!

When he is in the tunnel, he hears the spaceship making loud noises. Spider-Man keeps his hands, legs, and head still like a statue while he watches his movie.

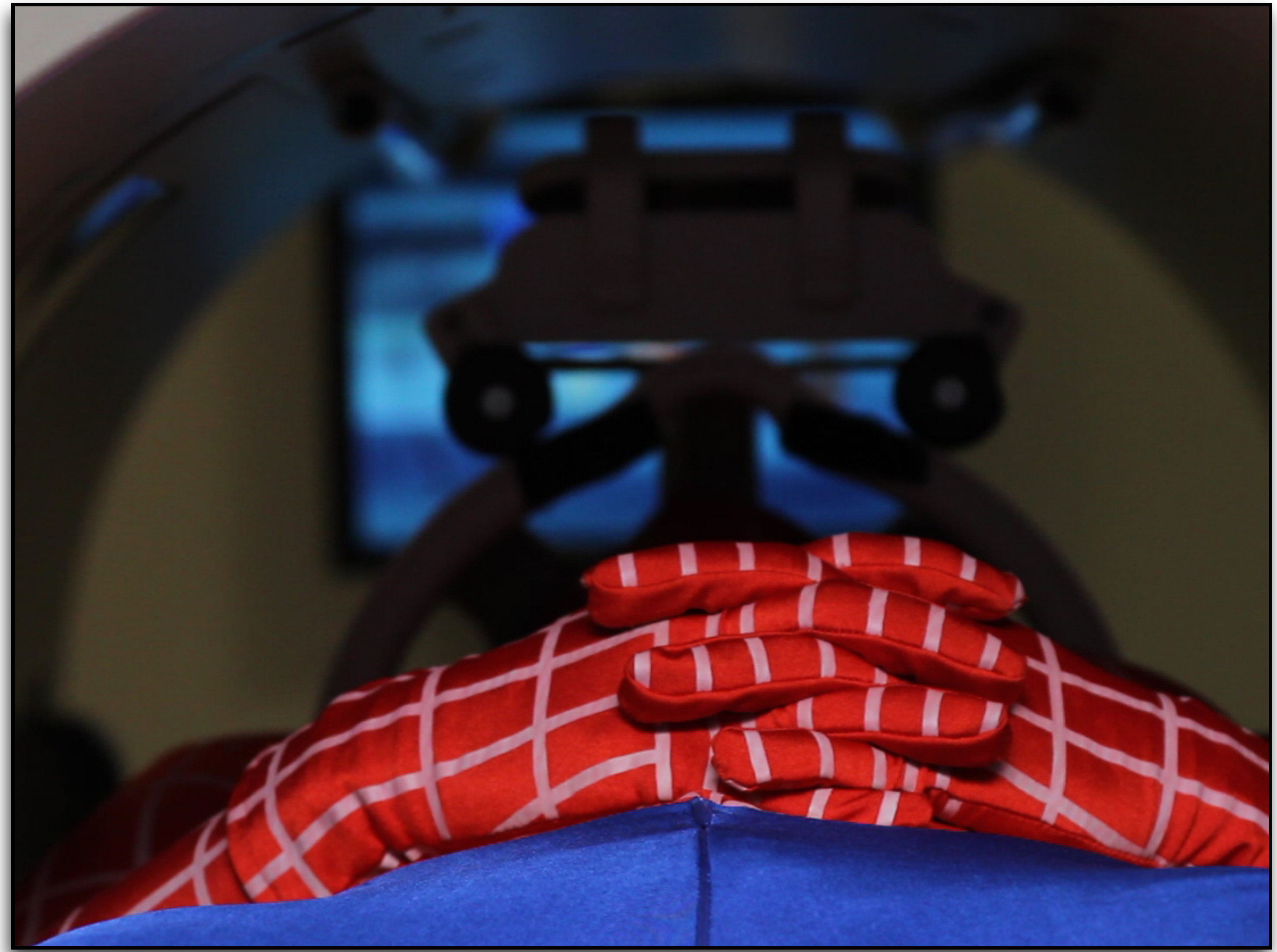

Spider-Man did a great job practicing for his MRI space mission! He gets a certificate which means he is ready for the real space mission!

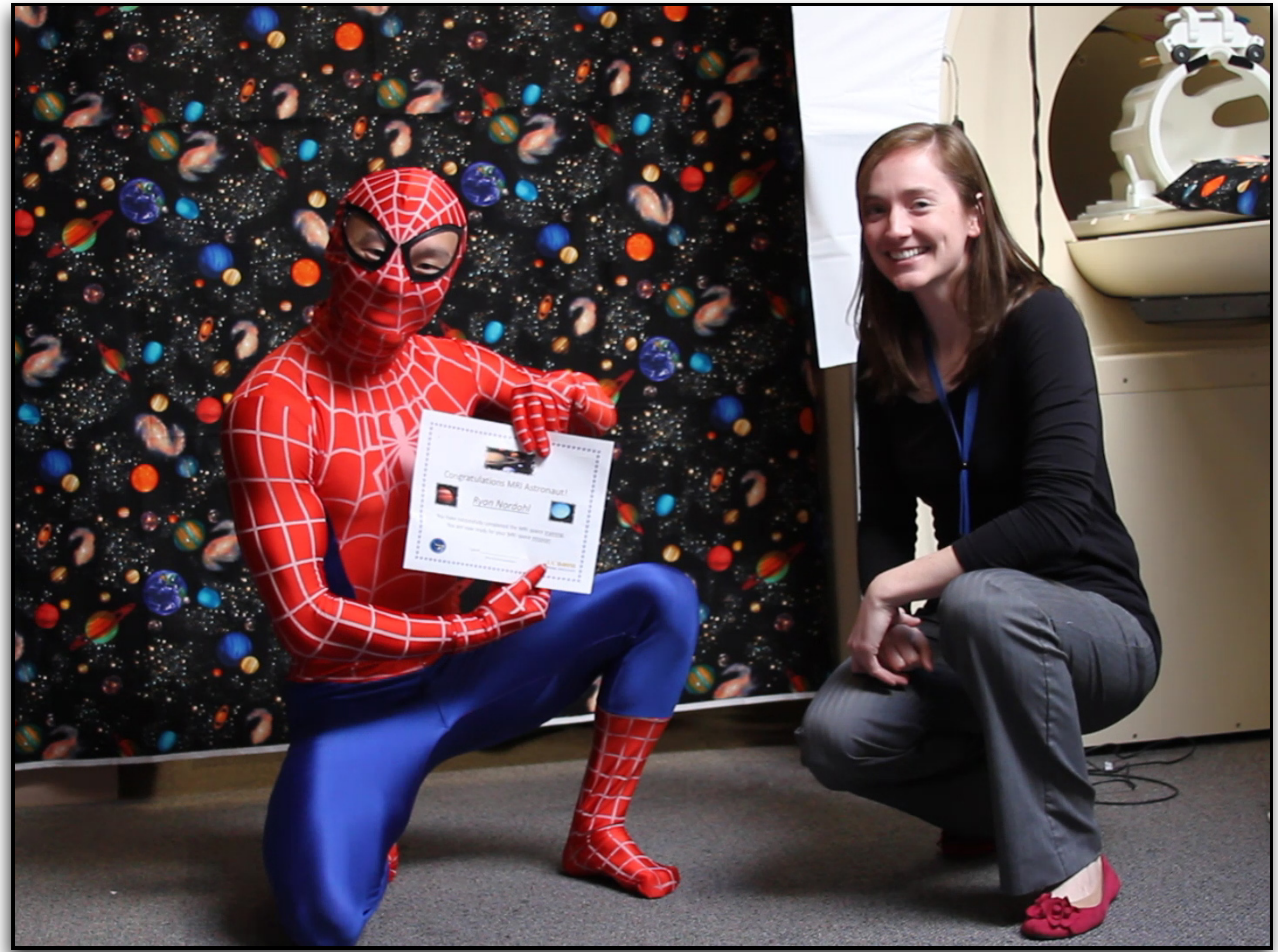

Spider-Man is excited to see the real MRI spaceship when he comes for his next visit. He knows what to do, and he is ready to stay still, so the spaceship can take pictures of his brain.

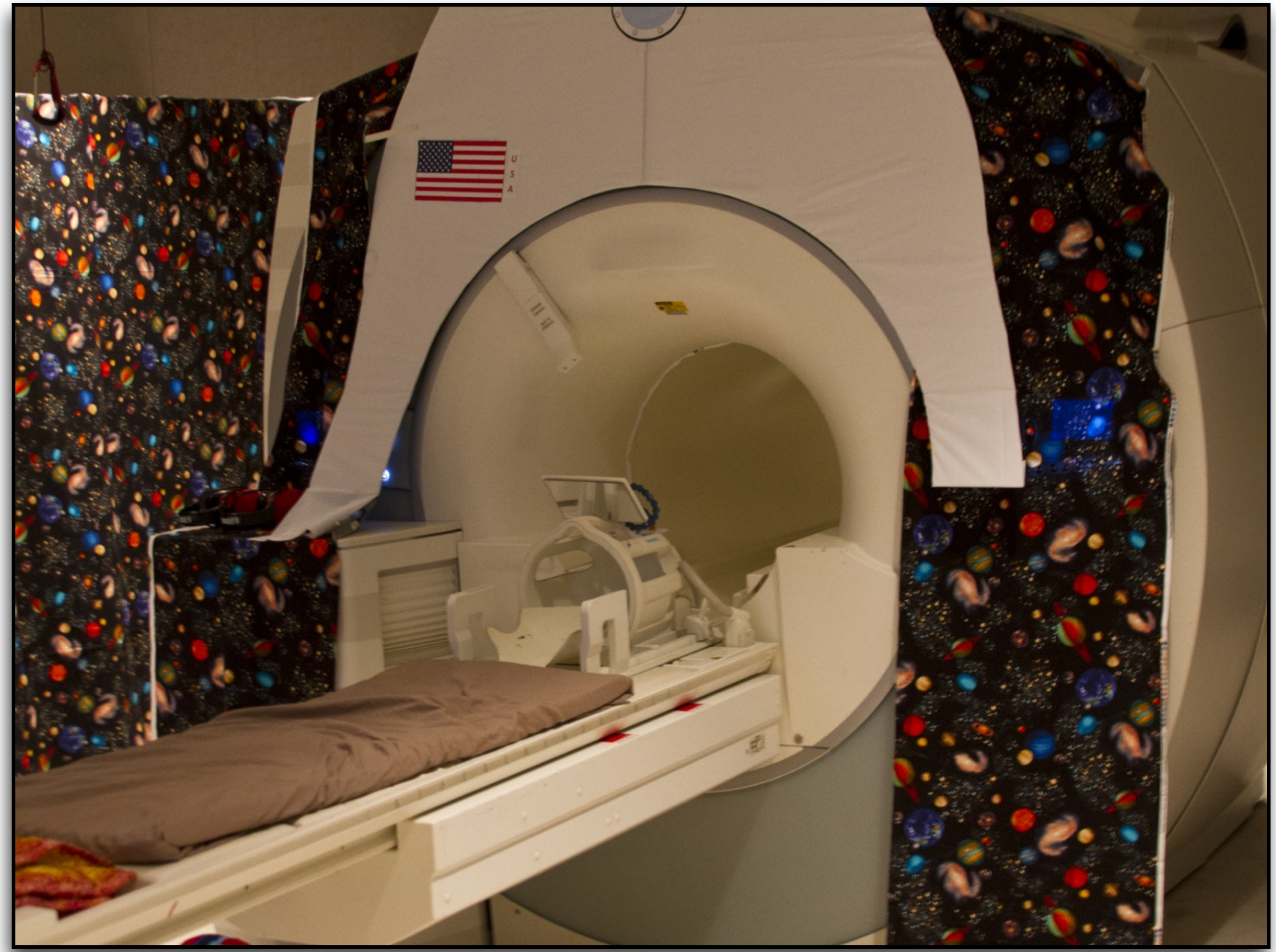

Spider-Man did awesome! Here is a picture of his brain. Now it is your turn Astronaut!

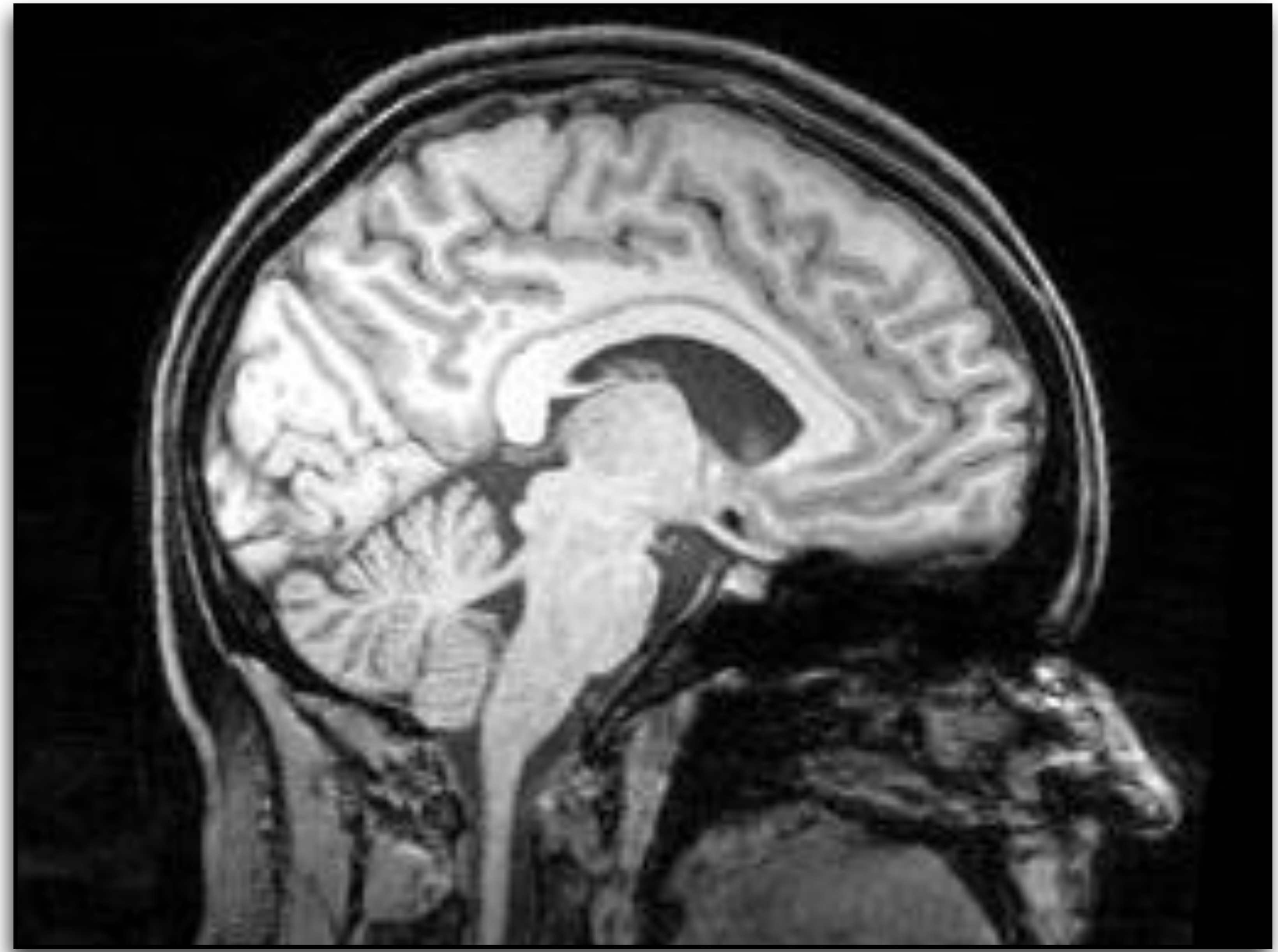

Supplement: Supplementary file 1 — Video story board. (PDF 7870 kb) [file 11689_2016_9154_MOESM1_ESM.pdf]
