# Supplementary material for: Methods for acquiring MRI data in children with autism spectrum disorder and intellectual impairment without the use of sedation
Source: J Neurodev Disord. 2016 May 5;8:20. doi: 10.1186/s11689-016-9154-9 (PMC4858915; doi:10.1186/s11689-016-9154-9)
Supplement: Supplementary file 2 — MRI Video Scan Script. (PDF 142 kb) [file 11689_2016_9154_MOESM2_ESM.pdf]

MRI Video Scan Script

Mock Session

Hi! I am __________ and I am here with Spiderman! We are here to show you how we can take pictures of your brain in our MRI spaceship! Spiderman is learning how to do the MRI with the help of an iPad. First, he looks at a picture of what he needs to do. Then, he does it himself. If he does it, he gets to play a game or watch a video on the iPad for a couple of minutes.

Step 1: First you will train with the practice MRI spaceship. This is the room where you will meet people from the space team and learn how to get ready for your MRI space mission.

Step 2: We will show you how to make the spaceship move, and you can even control it if you want to!

Step 3: Before you start your journey into space, you will climb onto the bed of the spaceship. You will get to see what it feels like when the bed moves. Spiderman really likes this part!

Step 4: Because the spaceship makes loud noises when it goes to space, we have a special pair of astronaut headphones for you to wear so it's not too loud.

Step 5: With your headphones on, you will lie down on the astronaut bed.

Step 6: You are now almost ready for take off! But you are missing a very important piece of your astronaut gear-your space helmet! When you are lying down with your headphones on, we will put a space helmet over your head. The helmet has a mirror so you can watch one of your favorite movies.

Step 7: 3-2-1 BLAST OFF!!!! You are ready to move into the space tunnel. One of the most important things to remember is to stay still during your trip into space.

Step 8: Once you are in the tunnel you will hear the spaceship making noises. Your only job is to stay still and watch your movie during the noises (just like Spiderman is doing). Each noise will last about 5 minutes and then you can take a break. Spiderman really liked watching a movie in the spaceship!!

Step 9: After you are done in the practice MRI spaceship, you will get your very own certificate that you finished space training!

Step 10: Congratulations astronaut! You are now ready for your real space mission!

MRI Session

Step 1: Next time you come to visit the spaceship, you will get to see the real MRI spaceship in a different room. Before you go into the new room, we will make sure you don't have anything in your pockets and are cleared for take off!

Step 2: First, we will show you pictures of the MRI spaceship on the iPad to remind you what you need to do.

Step 3: When you are ready for the real space mission, we will give you your own astronaut badge!

Step 4: With your badge on, you can now enter the room with the spaceship and prepare for your space mission.

Step 5: You won't be able to take the iPad on your mission, but you still get to watch one of your favorite movies.

Step 6: Remember, it's super important to stay still when the spaceship is making noises so we can get good pictures of your brain. If you want, you can see these pictures once you are done.

Step 7: Before you leave, you get to pick any prize you want from our toy basket.

Step 8: Great job astronaut! YOU DID IT! YOU COMPLETED THE SPACE MISSION!
