# Supplementary material for: Methods for acquiring MRI data in children with autism spectrum disorder and intellectual impairment without the use of sedation
Source: J Neurodev Disord. 2016 May 5;8:20. doi: 10.1186/s11689-016-9154-9 (PMC4858915; doi:10.1186/s11689-016-9154-9)
Supplement: Supplementary file 3 — MRI Video Scanning—Parent Handout. (PDF 33.6 kb) [file 11689_2016_9154_MOESM3_ESM.pdf]

MRI Video Scanning – Parent Handout

Mock Session

As part of the next visit for the Autism Phenome Project, we will be doing an MRI scan while your child is awake and watching a video. In order to prepare for the MRI scan, you and your child will come to the Imaging Research Center for a series of training sessions at our mock MRI scanner**.** We will work closely with you to do as many training sessions as necessary in the mock MRI room so that your child feels ready for the real MRI. These sessions are designed to be interactive visits where our team will help get your child accustomed to all of the steps that will take place during the actual MRI and will give you a chance to ask questions. You may remember seeing our mock MRI scanner when you came to one of your first visits with the Autism Phenome Project. The goal of that visit was to allow your child to have fun in the mock MRI room (your child might still remember playing with Thomas the Tank Engine!). While we still want these training sessions to be fun, we will try to focus more on helping your child complete the steps that will help him or her succeed in doing an MRI scan while awake. We have decorated both the mock and real MRI scanner using an outer space theme to help keep your child engaged.

Prior to the first training visit at the Imaging Research Center, one of our team members will call you to discuss an individualized plan for your child. We will have an iPad that we may use throughout the practice sessions. The iPad contains a slideshow of the MRI process with built in rewards/reinforcers. Each step will include a photo so that your child can see exactly what they will need to do to receive a reward. After they complete each step they will be allowed to select a video clip, an app to play, an activity, or a snack. We will talk with you beforehand to get your input on which rewards would be most effective for your child.

Step 1: To start, your child will enter the mock MRI room with you.

Step 2: We will encourage them to approach the bed and will show them how the bed can move in and out of the MRI tunnel.

Step 3: When comfortable, they will climb onto the bed with the help of you and a staff member. We can move the bed slightly while they are on it so they can get used to the motion.

Step 4: After they are able to sit on the bed, we will have them put on a pair of headphones. Attached to the headphones is a motion sensor that will help us gauge how much they are moving when the MRI sounds are playing (remember, laying still is the most important part!). As part of the training process we will use rewards and encouragement to help your child remain still for increasing periods of time.

Step 5: With the headphones on, they will lie down on the MRI bed.

Step 6: We will then lower a helmet down over their head. Attached to this helmet is a mirror so they can see the movie screen. Since they will not be able to hold onto the iPad beyond this step, we will transition to a movie of their choice.

Step 7: They will continue to stay in this position while watching a movie as we slowly move the bed into the tunnel. If the child is not comfortable moving all the way into the tunnel, we will give them plenty of time to adjust.

Step 8: Once your child is in the MRI tunnel with the headphones on and watching a movie clip, we will start to play the MRI sounds. We will practice with 5 sets of noises to simulate the real MRI sounds. Our

goal is to have your child lay still for 5 minutes at a time, for a total of 30 minutes. We recognize this is may be a long period of time for your child so we will take breaks when needed.

Step 9: Once your child has mastered each of the steps in the mock sessions, we will award them with an MRI Space Training Certificate!

Step 10: We will then schedule the real MRI scan during a day and time that works best with your schedule.

MRI Session

Step 1: When your child comes for the real MRI session, we will escort you to the MRI control room. This is where we will make sure everyone is metal free.

Step 2: Before they enter the MRI room (which will look very similar to the mock room), we can have your child review the practice steps on the iPad.

Step 3: We will present him or her with their very own MRI Astronaut Badge. This will help encourage them to feel less anxious about the MRI.

Step 4: Your child will then enter the MRI room, approach the bed, and complete the same steps that they mastered at the practice session.

Step 5: Although your child will not be able to take the iPad into the MRI room, we will play a movie on a large movie screen during the scan.

Step 6: If your child is able to lie still enough so we can collect the images, we will give you and your child a tour of their brain! (We will also send you a CD with a copy of the MRI)

Step 7: After the scan, your child can pick out any prize and you will receive compensation for your time.
