# Supplementary material for: Methods for acquiring MRI data in children with autism spectrum disorder and intellectual impairment without the use of sedation
Source: J Neurodev Disord. 2016 May 5;8:20. doi: 10.1186/s11689-016-9154-9 (PMC4858915; doi:10.1186/s11689-016-9154-9)
Supplement: Supplementary file 4 — T scores and age equivalent scores for the DAS subscales. (PDF 96.1 kb) [file 11689_2016_9154_MOESM4_ESM.pdf]

Supplementary Table 1. T-scores and age equivalent scores for the DAS subscales

Partici-pant	Recall of Designs		Word Definitions		Verbal Similiarites		Sequential and Quantitative Reasoning		Matrices		Pattern Recognition	
	t-score	Age equiv	t-score	Age equiv	t-score	Age equiv	t-score	Age equiv	t-score	Age equiv	t-score	Age equiv
**1**	40	85	25	58	26	67	27	61	27	49	32	64
**2**	10	58	10	58	10	58	17	58	10	43	10	31
**3**	45	100	31	70	24	67	33	76	31	64	42	94
**4**	10	58	10	58	10	58	16	58	10	43	10	31
**5**	10	58	10	58	10	58	28	67	33	73	10	31
**6**	20	61	10	58	10	58	22	58	26	52	24	55
**7**	10	58	10	58	10	58	15	58	23	43	57	147
**8**	26	79	10	117	10	88	25	117	21	100	28	82
**9**	30	58	43	64	28	58	42	67	37	79	33	58
**10**	10	58	21	58	10	58	23	58	31	43	22	61
**11**	10	76	10	58	10	58	10	70	10	43	23	31
**12**	29	91	10	58	10	58	24	94	17	76	10	111
**13**	33	70	10	58	10	58	32	67	29	43	39	64
**14**	53	135	45	106	53	129	66	177	78	213	51	123
**15**	46	123	62	183	63	216	46	129	50	141	46	123
**16**	53	153	47	129	55	165	43	117	54	171	54	159
**17**	67	216	28	88	35	106	44	135	51	171	68	216

Participant numbers match those depicted in Tables 1 and 2 of main text.

Participants 1-13 have IQs in the range of ID
