# Supplementary material for: Methods for acquiring MRI data in children with autism spectrum disorder and intellectual impairment without the use of sedation
Source: J Neurodev Disord. 2016 May 5;8:20. doi: 10.1186/s11689-016-9154-9 (PMC4858915; doi:10.1186/s11689-016-9154-9)
Supplement: Supplementary file 5 — Supplementary materials: Individual narratives. (PDF 75 kb) [file 11689_2016_9154_MOESM5_ESM.pdf]

Supplementary materials: Individual narratives

Participant 4 (BN)

BN was 9 years; 9 months old at the time of the scan, with an IQ of 41.1 and ADOS total score of 19. At his behavioral assessment, he became agitated by requests to respond and displayed rapid head banging and aggressive behavior toward his caregivers. He used one sound to indicate ‘no’ and did not use any spontaneous gestures or make eye contact. BN occasionally copied actions with objects but did not show spontaneous play actions.

BN was visibly upset when he arrived for his first mock session at the Imaging Research Center and was initially resistant to following instructions. To help **pair** the mock environment with reinforcement, the behavior analyst greeted him with his preferred items (sensory bin and iPad) in the lobby. Once he calmed down and complied with simple one-step instructions, the behavior analyst used **behavior momentum** to transition BN to the mock scanner room where he was eager to explore. The behavior analyst used the iPad, preferred video, and preferred snack as contingency-based reinforcers to encourage him to approach and sit on the mock scanner bed. This was accomplished using the **Premack principle**, using first-then statements such as “Okay first sit on the bed, and then iPad.” His preferred video played on the monitor at the end of the mock scanner bed, which served as the reinforcer for putting on headphones, lying down, and pulling the headcoil down so that he could view and hear his video. Once BN was able to lie down on the scanner bed, he was able to lie still inside the scanner with the weighted blanket on him and his parents massaging his feet. During this initial session, the behavior analyst decided not to introduce the motion potentiometer and gradient sounds. BN was able to lie in the scanner for 5 minutes during the first mock session.

One week later, BN returned for a second mock session, eager to return into the mock scanner to watch his video. He easily transitioned onto the bed wearing silicone earplugs and headphones and into the mock scanner to watch his preferred video. Once he was settled into

the scanner, the motion potentiometer was turned on and was very effective in decreasing his head motion. BN lied in the mock scanner for 20-25 minutes covered with the weighted blanket and his parents massaging his feet. The gradient noises were introduced at the lowest volume and systematically increased to max volume as he showed success with lying still.

BN returned for the MRI scan session one week later. Upon arrival he smiled and seemed very excited to be at the IRC and to see the MRI team. In the 3T scanner room he cooperated with the placement of silicone earplugs, wearing headphones, and lying on the scanner bed. Once he was in the scanner, we conducted a 10-minute training session to remind him to be still. We utilized the **visual countdown timer** displayed on the bottom of the video screen and gave **verbal reminders** to remain still for the amount of time remaining on the timer. Because BN responded well to the motion potentiometer in the mock scanner, we created a similar system in the 3T scanner room in which the team manually blacked out the video contingent on his movement. Throughout the scanning session, the behavior analyst stood outside the bore and watched for movement and signaled the team to black out the screen. Each time the screen was blacked out, BN responded by decreasing his movement. During this time, he was covered with the weighted blanket while his parents massaged his feet. The first four attempts at the MPRAGE did not reach acceptable motion criteria. BN was then given a break with his preferred snack. When he returned to the scanner, greater efforts were made to ensure his comfort. We put a pillow under his knees and made sure his head was in a comfortable position supported by memory foam. The behavior analyst also increased the frequency of **verbal reminders** and praise. As a result, NB was able to successfully remain still during this 5th attempt at the MPRAGE, which passed the quantitative assessment for motion criteria. Immediately following the successful MPRAGE, the DTI was attempted and successfully acquired on the first attempt, passing the motion criteria.

Participant #5 (CO)

CO was 9 years 10 months at the time of the scan, with a current IQ of 47.7 and ADOS total score of 19. At her behavioral assessment, she had an anxious manner and remained withdrawn from unfamiliar people and places. When agitated, CO cried and turned away. She used word approximations for no/yes and these were often accompanied with nonverbal gestures. CO indicated her preference for objects with a point and occasionally paired this with eye contact. She smelled every object that she touched and enjoyed solitary activities but would look to her caregiver to smile at times.

CO arrived at the IRC with her father initially very calm and the behavior analyst reviewed the **visual storyboard** that CO had brought with her. Upon entering the mock scanner room, however, she became very anxious as evidenced by vocal protesting and refusing to step close to the mock MRI bed. Her father shared that she had a fear of medical environments. She sat down in a chair against the wall. Using her preferred snacks, the process of shaping began with the behavior analyst moving her chair two inches closer to the mock bed then stopping to allow her to get comfortable and have some of her snack following the small movement. This process was repeated over the next twenty minutes until she had her feet on the stool below the mock bed. Throughout this process, the behavior analyst was **pairing** both herself and the room with reinforcement, and desensitizing CO to the environment. The behavior analyst then tried to have CO stand on the stool (in order to get on the table), which she attempted once, very briefly, on her own with reinforcement and first/then statements (first put feet up, then more snack). Twice the behavior analyst attempted to physically prompt her to stand on the stool, but she was very resistant. Her father **modelled** sitting on the table, and eventually, with priming (being shown pictures from the **storyboard**) and a **countdown** from ten (a calming technique used at home); he was able to seat CO on the mock bed. She ate more of her snack and was given time to adjust to sitting on the table. Once calm, she was physically

prompted to place her feet completely on the table. The headphones were placed on her with the audio from her preferred video playing, which she was able to tolerate.

Once comfortable with her feet on the table, she was again primed using the visuals, given a count down and prompted to lie all the way down. She was then given a break, during which time the behavior analyst twice showed her that the table would move. Upon getting back onto the table, laying down with headphones on, she was moved partially into the MRI bore. She was given her preferred snack placed on her chest as comfort and again a **countdown** from ten helped calm her. She was quickly able to stay in the mock MRI, without reinforcers, for over 5 minutes. The MRI gradient sounds were introduced and quickly increased in volume as she was able to tolerate the gradient noises with little problem, except that she would startle at the onset of the sounds. Inside the mock scanner, it was clear that she was not using the mirror mounted on the head coil to watch the video, so the team discussed other options that might work for her. Her father mentioned that she has a favorite music artist, so we switched over to playing music from this artist instead of watching the video. The behavior analyst felt CO was ready to be transitioned to the 3T environment, and that it would be optimal to do so within the same day given that her level of anxiety was slowly beginning to dissipate. The process of desensitization seemed to be working, as CO was starting to trust that no medical equipment (needles, etc) were going to be used on her.

After a short break, CO entered the 3T suite and, although hesitant, was able to complete all the steps with relative ease and minimal prompting. The **visual timer** was set up on the screen and she listened to her favorite artist in the scanner. The first four MPRAGE attempts did not meet the QA motion criteria, so adjustments were made to the environment. First, it was noted that the visual timer seemed to be more of a distraction that caused her to look down at the screen, causing head movement, so it was removed. The album of her favorite artist was being played from YouTube, which was interrupted with advertisements between songs. This also led to increased movement until a continuous recording of the entire

album was initiated. An additional blanket was placed over the weighted blanket to increase her comfort level inside the bore. The behavior analyst and the child’s father **modeled** being still and pointed out specific body positioning (“Wow, Dad’s face is so still! He is not moving his arms!”). Also, because CO exhibited a startle response at the onset of each MPRAGE, the scanner operator made adjustments by starting an MPRAGE sequence and allowing it run for 10 seconds, then in quick succession, stopping that sequence and starting a new MPRAGE sequence. The MPRAGE was successful on the fifth attempt. The DTI sequence was not attempted.
